# PROTECTING COGNITION AND INCREASING EQUITY THROUGH “COGNITIVE IMPACT ASSESSMENTS”

**DOI:** 10.1093/geroni/igad104.0230

**Published:** 2023-12-21

**Authors:** Anja Leist, Ariane Bertogg, Skerdi Zanaj

**Affiliations:** University of Luxembourg, Esch-sur-Alzette, Luxembourg; Ludwig-Maximilians-Universitaet Muenchen, Munich, Bayern, Germany; University of Luxembourg, Luxembourg, Grevenmacher, Luxembourg

## Abstract

Today’s societies are characterized by increasing complexity, in which unimpaired cognitive performance is vital to navigate and participate, as well as to retain independence at older ages. Addressing the challenge of improving population brain health has mostly been considered from a dementia risk reduction perspective as identified by the Lancet Commission on dementia. However, risk factors with immediate harm to cognitive functioning, such as shift work or insufficient sleep are currently not explicitly accounted for. As exposure to these risk factors accumulates across the life course, we can expect substantial consequences for later-life brain health. We argue that, in policy and practice, systematic measures should be introduced to protect individuals from harms to their everyday cognitive functioning, and enable them to optimize cognitive performance throughout the life course. On behalf of the international multidisciplinary BRAINHEALTH-POLICY group, we explore the concept of a ‘cognitive footprint’ of policies with exemplars from current legislation and policy that either implicitly affect cognition, or explicitly address effects on individuals’ cognition. We suggest recommendations for systematic pre-intervention ‘cognitive impact assessments’ in fields such as medicine, where drug trials should include cognition as important secondary endpoint, and work, where labour laws should explicitly account for possibly harmful effects of certain work conditions to cognition. Besides immediate protective effects, we expect ‘cognitive impact assessments’ to have potential for long-term cognitive benefits on a population level as well as improve brain health equity by enabling individuals to reach optimum cognitive performance, with associated wider health benefits.

